# Biomimetic Soft Actuator with Deformation and Motion Driven by Near-Infrared Light

**DOI:** 10.3390/polym17101315

**Published:** 2025-05-12

**Authors:** Mei Li, Yubai Ma

**Affiliations:** 1 College of Materials Science and Engineering, Chongqing University, Chongqing 400044, China; 15156215827@163.com; 2 School of Chemistry and Chemical Engineering, Chongqing University, Chongqing 400044, China

**Keywords:** soft actuators, MWCNT/LCP nanocomposites, NIR driven, deformation and motion, liquid crystal to isotropic phase transition

## Abstract

Restricted by the inherent low sensitivity of materials and complex integration technology, it is difficult for existing soft actuators (s-actuators) to simultaneously possess the advantages of flexibility, fast response, and simple manufacturing, which greatly limits their practical applications. Herein, a stretchable (ε = 200%) nanocomposite film capable of deformation and motion driven by near infrared light (NIR) was developed using multi-walled carbon nanotubes (MWCNTs) as the light absorption–photothermal conversion nanonetwork, and liquid crystal polymer (LCP) as an elastic matrix featured reversible phase transition. Furthermore, s-actuators with various deformation and motion modes have been realized employing MWCNT/LCP nanocomposite film. Based on the mechanism that photothermal-effect-regulated liquid crystal–isotropic phase transition in LCP can induce macroscopic deformation of nanocomposites, MWCNT/LCP s-actuators have completed a series of complex deformation and motion tasks such as opening the knot, “V”-shape reversible deformation (30 s per cycle), the “spring” rotating and unfolding, imitating a “caterpillar” walking in a straight line (the average speed is 13 s/mm), etc. This work provides an effective strategy for the intelligent development of s-actuators.

## 1. Introduction

Soft actuators (S-actuators) are biomimetic systems assembled from flexible smart materials [1,2,3]. Compared with electrically driven rigid actuators, s-actuators can be driven by various stimuli such as light, electricity, heat, magnetism, and chemicals based on the intrinsic characteristics of smart materials [4,5,6], granting them advantages of remote, noncontact interaction, and controllable deformation. In addition, they can provide more flexible shape changes and safer and more comfortable human–computer interaction. Therefore, they show fascinating application potentials in bionic robotics [7,8,9], soft grippers [10,11,12], and biomedical systems [13,14]. In recent years, flexible actuator materials, as one of the most cutting-edge research hotspots in the field of new materials, have already achieved basic actuation functionalities [15,16,17]. However, existing s-actuators are difficult to simultaneously meet the requirements of flexibility, fast response, and simple manufacturing processes due to the inherent low sensitivity of materials and the processing technology of integrating multiple materials into special micro-nano structures [18,19,20]. These limitations severely restrict the practical application of s-actuators. In order to achieve excellent comprehensive performance, a variety of actuating materials have been explored, such as hydrogels, shape memory polymers, and liquid crystal polymers. However, hydrogels can deform or move only in the presence of a large amount of water, and their low elastic modulus leads to slow actuation speed (response time is usually on the order of minutes) [21,22,23]; shape memory polymers, which generally are driven by thermal effects, suffer from limited deformation and poor reversibility due to the slow cooling rate preventing each cycle from returning to the initial temperature [24,25,26]; and it is still very challenging for liquid crystalline polymers to readily process them into s-actuator capable of versatile locomotion [27,28,29]. Therefore, there is an urgent demand for a flexible smart material that can be facilely processed into actuators capable of fast response and large deformation to promote the development of s-actuators.

Herein, a novel smart nanocomposite capable of shape morphing and motion in response to NIR was developed by dispersing the MWCNTs into an elastic matrix of LCP uniformly, in which the MWCNTs were acted as a nano-network for light absorption, photothermal conversion, and conduction [30,31,32,33], while LCP had movable molecular chains. Under NIR irradiation, the photothermal conversion of MWCNTs can cause a phase transition of LCP from liquid crystal to isotropic phase that is conducive to the movement of molecular chains, resulting in macroscopic deformation and movement of the MWCNT/LCP nanocomposites. Several s-actuator models and corresponding motion modes were designed employing MWCNT/LCP. By precisely controlling the position and time of NIR irradiation, MWCNT/LCP actuators were driven to complete a series of shape morphing and motion tasks, mimicking the behavior of creatures in nature, such as opening a knot, “stretch-restoration” reversible deformation of “V”-shape (30 s per cycle), the “spring” rotating and unfolding, imitating a “caterpillar” to walk in a straight line (the average speed is 13 s/mm), an “insect” crawling and rolling, etc. Furthermore, MWCNT/LCP nanocomposite films exhibited superior flexibility and stretchability (*ε* = 200%). With the vivid deformation and motion, wearability, and being easy to fabricate, the innovative MWCNT/LCP s-actuators take a significant step forward toward their potential application in soft robotics.

## 2. Materials and Methods

### 2.1. Materials

MWCNTs (diameter: 8~15 nm; length: 50 μm; purity: 98%) were purchased from Beijing Deke Daojin Science and Technology Co. Ltd. (Beijing, China); *p*-nitroaniline (purity: 99%), hydrochloric acid (content: 36.0~38.0%), sodium nitrite (analytical grade), sodium dodecyl sulfonate (analytical grade), hexamethylenediamine (116.2 kDa), toluene (analytical grade), and *N*,*N*-dimethylformamide (analytical grade) were purchased from Shanghai Wo Kai Biological Technology Co. Ltd. (Shanghai, China); 1,4-bis- [4-(6-acryloyloxyhexoxy) benzoyloxy]-2-methylbenzene (RM82, 672.76 kDa, purity: 99%) was purchased from Beijing Bayi Space Time LCD Technology Co., Ltd. (Beijing, China); Irgacure 369 (I369) was purchased from Shanghai Houcheng Fine Chemical Co., Ltd. (Shanghai, China).

### 2.2. Surface Functionalization of MWCNTs

In order to improve the dispersion of MWCNTs in organic solvents, surface functionalization of MWCNTs was carried out using *p*-nitroaniline diazonium salt. First, 35 mmol *p*-nitroaniline (purity = 99%) was completely dissolved in a mixture of 150 mL deionized water and 8.8 mL concentrated hydrochloric acid by magnetic stirring. Then, 2.5 g of sodium nitrite was added, and the mixture was magnetically stirred at 0~5 °C for 40 min, and the product solution was the desired *p*-nitroaniline diazonium salt, which would be used for surface functionalization of MWCNTs. Subsequently, 10 wt% sodium dodecyl sulfonate was added to 1.0 g MWCNTs as a surfactant and dispersed ultrasonically in 800 mL deionized water. The freshly prepared *p*-nitroaniline diazonium salt was then slowly added dropwise into the dispersion, accompanied by ultrasonication (Ultrasonic Homogenizer JY92-IIN, Shanghai Xiwen Biotech. Co., Ltd., Shanghai, China) and magnetic stirring at 10 °C. After that, the mixture continued to be magnetically stirred and ultrasonicated for 5 h at 38 °C in air. The resulting MWCNTs suspension underwent filtration, soxhlet extraction (48 h), and freeze-drying successively and, finally, obtained well-dispersed MWCNTs. The ultrasonication power used in this procedure was 400 W. The scanning electron microscopy (SEM) images of surface-functionalized MWCNTs with scales of 200 and 100 nm are shown in Figure S1a,b.

### 2.3. Preparation of the MWCNT/LCP Nanocomposites

**Figure 1:**
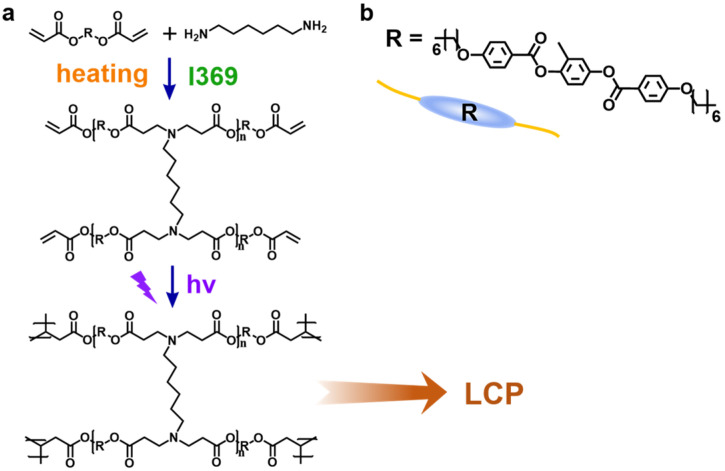
(**a**) The synthesis route and molecular structure of LCP; (**b**) chemical structural of the mesogenic unit R.

The preparation of MWCNT/LCP nanocomposites was carried out in two steps: (1) LCP was prepared by mixing RM82 (672.76 kDa) and Hexamethylenediamine (116.2 kDa) at a molar ratio of 2:1. Toluene (40 mL·g^−1^) was then added into the reaction mixture, which was heated (IKA C-MAG HS7, Germany) to 80 °C in a nitrogen atmosphere under mechanical stirring (IKAEUROSTAR 20 digital, Germany) at 500 r/min. Then, I369 was added as a photo-initiator with a mass of 1.5 wt% of the reactant and reacted at 80 °C for 6 h to obtain the desired LCP. Figure 1a shows the synthesis route and molecular structure of LCP, and Figure 1b shows the chemical structural of the mesogenic unit R. Figure S1c shows the SEM image of the surface of LCP film. (2) A certain amount of surface-functionalized MWCNTs (m_MWCNTs_ = 2 wt% m_LCP_) was dispersed in *N*,*N*-dimethylformamide by ultrasonication (40 min) to form a uniform and stable MWCNTs suspension, which was then mixed with the newly prepared LCP solution for another 30 min of ultrasonication, and then the solvent was removed by rotary evaporation (IKA RV8, IKA Rotary Evaporators, Staufen, Germany) until 10 mL of the concentrated mixture remained. Subsequently, the remaining product mixture continued to be ultrasonicated for 20 min to ensure the MWCNTs were uniformly dispersed. Finally, the product mixture was poured into a petri dish and irradiated with 365 nm ultraviolet light for 30 min while heating on a hot plate (IKA RCT basic, IKA Rotary Evaporators, Staufen, Germany) at 85 °C to activate photo-crosslinking. The product mixture was then heated at 80 °C for at least 12 h to form an MWCNT/LCP nanocomposite thin film with a thickness of 0.5 ± 0.1 mm. It should be noted that all ultrasonication used in this procedure had a power of 300 W at room temperature.

### 2.4. Fabrication of the MWCNT/LCP S-Actuators

Several deformation and motion modes were designed, such as opening the knot, “V”-shape reversible deformation, the “spring” rotating and unfolding, imitating a “caterpillar” walking in a straight line, “flower” in bloom, imitating “insects” crawling and rolling, etc. For each mode, the prepared MWCNT/LCP nanocomposite films (thickness: 0.5 ± 0.1 mm) were cut into strip samples with widths ranging at 1~3 mm, and then the samples were shaped into predesigned s-actuator models for deformation and motion performance measurement.

### 2.5. Deformation and Motion Performance Measurement of MWCNT/LCP S-Actuators

Each s-actuator model was irradiated with 808 nm NIR (MW-IR-1064/200 mW 16090762) at room temperature in air, driving them to complete specific deformation and motion tasks. As for the time of NIR on/off, continuous or periodic irradiation should be selected according to the deformation and motion modes to be achieved. The local temperature changes of MWCNT/LCP s-actuators caused by NIR were measured by a K-type thermocouple connected to a temperature logger (Pico Technology USB TC-08, Eaton Socon, UK). Actuation stress during reversible deformation of the “V” s-actuator was measured by a strain gauge (KFGS-2-350-D1-23, KYOWA, Toyo, Japan) connected to a dynamic strain testing system (DH3818N, Donghua Testing Technology Co., Ltd., Taizhou, China).

### 2.6. Characterization

The dispersion of MWCNTs in nanocomposites was observed through SEM (GeminiSEM300, Carl Zeiss, Oberkochen, Germany). The chemical structure of LCP and MWCNT/LCP nanocomposites was analyzed by Fourier transform infrared spectroscopy (FTIR, VERTEX 70, Bruker, Germany). Absorbances of LCP and MWCNT/LCP nanocomposites were investigated by ultraviolet–visible light–near infrared (UV-Vis-NIR, UV-3600 Plus, Shimadzu, Kyoto, Japan) spectroscopy at room temperature. Phase transition of LCP and MWCNT/LCP nanocomposites was characterized by the differential scanning calorimetry (DSC, Diamond DSC, PerkinElmer Instruments, Shanghai, China) and X-ray diffraction (XRD, x’pert3 powder, PANalytical B.V., Almelo, The Netherlands). It is worth noting that the XRD testing of LCP at 5 °C and 32 °C was carried out using a JK-CH600190-XRD cold and hot stage. The samples were cooled to 5 °C by liquid nitrogen cooling and heated to 32 °C by resistance heating. The thickness of MWCNT/LCP films was measured using a thickness gauge (033004, EXPLOIT Co., Ltd., Rizhao, China).

## 3. Results

### 3.1. Overview of the Functions and Properties of MWCNT/LCP Nanocomposites

**Figure 2:**
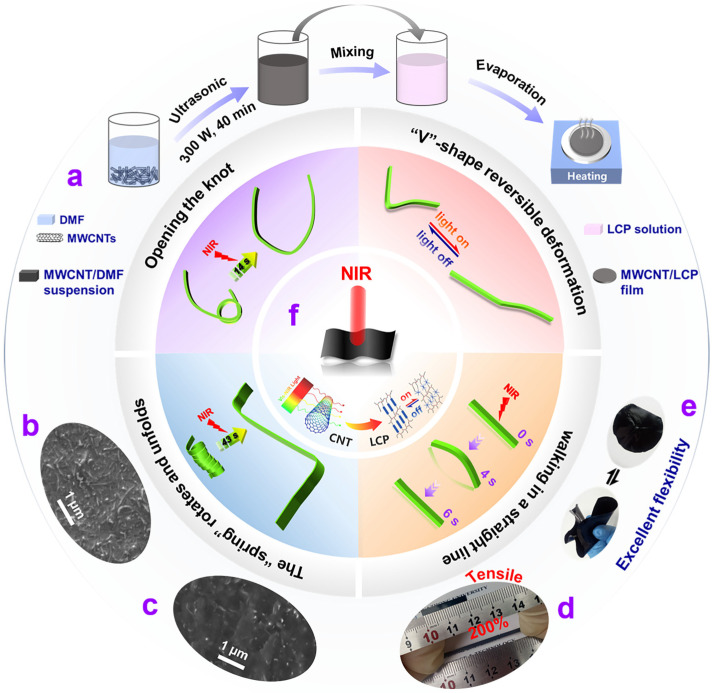
(**a**) Schematic diagram of the fabrication process for the MWCNT/LCP nanocomposites; scanning electron microscopy (SEM) images of (**b**) surface and (**c**) cross-section of 2 wt% MWCNT/LCP film; optical photograph of the 2 wt% MWCNT/LCP films (thickness: 0.5 mm) show superb (**d**) stretchability (*ε* = 200%) and (**e**) flexibility; (**f**) schematic diagrams of several typical deformation and motion modes implemented by MWCNT/LCP s-actuators under continuous or periodic NIR irradiation.

MWCNT/LCP nanocomposites proposed here were employed for s-actuators that can imitate diverse motion modes found in nature, and their schematic preparation process is illustrated in Figure 2a. Mesogens were used as the light-sensitive phase transition units, hexanediamine and I369 as the chain extender and photo-crosslinking agent, respectively, and they crosslinked into LCP by exposure to ultraviolet (UV). The surface-functionalized MWCNTs were uniformly dispersed in fresh LCP solution by ultrasound, and the MWCNT/LCP films were obtained by heating evaporation.

Compared with carbon nanomaterials such as graphene and fullerenes, carbon nanotubes (CNTs) have advantages such as one-dimensional structure with an aspect ratio of 10^2^~10^3^ (Figure S1a,b), higher thermal conductivity, and lower cost, which endow them with extremely high tensile strength and flexibility, as well as excellent photo-thermal performance [34,35,36], fully meeting the design requirements of actuating materials with superior flexibility, tensile property, and thermally induced deformation in this study.

As for CNTs, the aspect ratio of single-walled carbon nanotubes is greater than 10^3^, making it exceptionally difficult to surface functionalize and evenly disperse them in the polymer matrix. Moreover, they are very expensive. Based on the above performance and cost advantages, MWCNTs are selected as functional fillers in this study.

The chemical structure of LCP and MWCNT/LCP nanocomposites was analyzed by the FTIR (Figure S2 and Table S1 in Supplementary Materials), confirming that the functional groups of MWCNT/LCP were completely consistent with the molecular structure designed in this study.

The microstructure of MWCNT/LCP films was studied via SEM. As shown in Figure 2b,c and Figure S3 (Supplementary Materials), MWCNTs were uniformly distributed both on the surface (Figure 2b and Figure S3a) and inside (Figure 2c and Figure S3b) of the film, forming a dense and uniform network structure, which could absorb NIR and convert light energy into thermal energy [30,31,32,33], causing the temperature of MWCNT/LCP to rapidly increase; subsequently, there was a transition from liquid crystal phase to isotropic phase at the micro level, so the rod-shaped mesogenic unit was destroyed, and mechanical deformation and motion of MWCNT/LCP occurred at the macro level [37,38]. Figure 2d,e show the excellent stretchability and flexibility of MWCNT/LCP films. The tensile strain of the 2 wt% MWCNT/LCP film with a thickness of 0.5 mm can reach 200% (Figure S4a). Additionally, the film can be arbitrarily curled and shape morphed then rapidly restored after the external force disappears, as shown in Figure 2e and Figure S4b (Supplementary Materials). The intelligent characteristics of MWCNT/LCP that could automatically deform and motion like creatures in nature under NIR irradiation are shown schematically in Figure 2f, demonstrating that MWCNT/LCP could complete intense deformation and motion tasks such as opening the knot, “V”-shape reversible deformation, the “spring” rotating and unfolding, and imitating a “caterpillar” walking in a straight line.

### 3.2. The Mechanism of MWCNT/LCP S-Actuators Deformation and Movement

**Figure 3:**
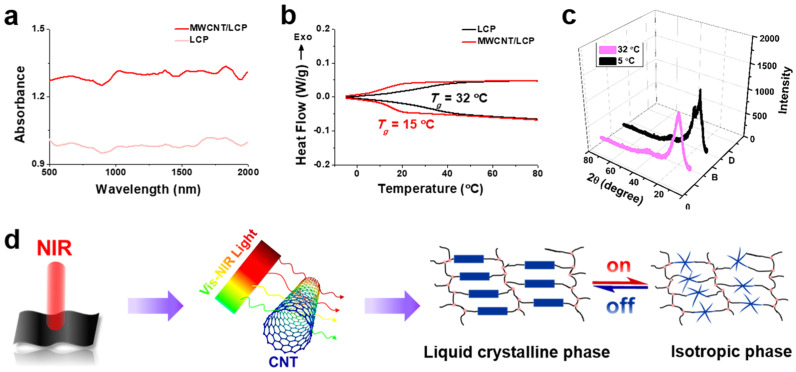
(**a**) UV−Vis−NIR absorbance spectroscope of LCP and 2 wt% MWCNT/LCP nanocomposite; (**b**) DSC curves of LCP and 2 wt% MWCNT/LCP nanocomposite (heating and cooling rate: 5 °C/min); (**c**) XRD patterns of LCP at 5 and 32 °C in air; (**d**) schematic diagram of the working mechanism of MWCNT/LCP s-actuator deformation and motion driven by NIR.

The macroscopic deformation and motion of MWCNT/LCP s-actuators are caused by the destruction of the rod-shaped mesogenic unit of the LCP at the micro level. Figure 3a shows the UV-Vis-NIR absorption spectra of LCP and 2 wt% MWCNT/LCP nanocomposite, which is used to characterize the light absorption of the materials. The results show that LCP has weak absorption for visible and NIR light, while MWCNT/LCP nanocomposite doped with 2 wt% MWCNTs significantly improved their absorption, indicating a superior light absorption of MWCNTs. In fact, MWCNTs form a uniform network for light absorption, photo-thermal conversion, and thermal conduction in MWCNT/LCP nanocomposites, which convert the absorbed light energy into heat when exposed to NIR, and then transmit this heat together with the heat from infrared radiation to the MWCNT/LCP, causing its temperature to rise rapidly. According to the author’s previous research [30,31,32,33], the photothermal conversion efficiency of MWCNTs in the elastic polymer matrix is approximately 78.5%. When the temperature reaches the glass transition temperature (*T_g_*) of LCP, the LCP matrix of nanocomposites undergoes a transition from liquid crystal phase to isotropic phase at the micro level. That is, during the continuous heating process (NIR on), the rod-shaped mesogenic units are gradually destroyed and become isotropic phase [37,38]. The *T_g_* of LCP and 2 wt% MWCNT/LCP were measured by DSC at 32 and 15 °C (Figure 3b), respectively, demonstrating that both materials undergo a reversible phase transition during the heating/cooling process. The microscopic phase transition of LCP from liquid crystal to isotropic phase can also be confirmed by XRD patterns (Figure 3c). Figure 3c shows that the sharp diffraction double peaks transform into a single peak when the LCP is heated from 5 to 32 °C. Double peaks are typical crystal diffraction peaks, while single peaks are amorphous diffraction peaks [39,40]. The NIR-induced microscopic phase transition of materials provides a liquid environment for molecular chains to move easily, namely, molecular chains of nanocomposites are bound in the liquid crystal state but move actively when the mesogenic unit is destroyed [41,42,43], which manifests macroscopically as changes in the morphology and position of MWCNT/LCP s-actuators. Figure 3d is a schematic diagram of the working mechanism for the deformation and motion of MWCNT/LCP s-actuators under NIR irradiation.

### 3.3. Performance of MWCNT/LCP S-Actuators Deformation and Motion

**Figure 4:**
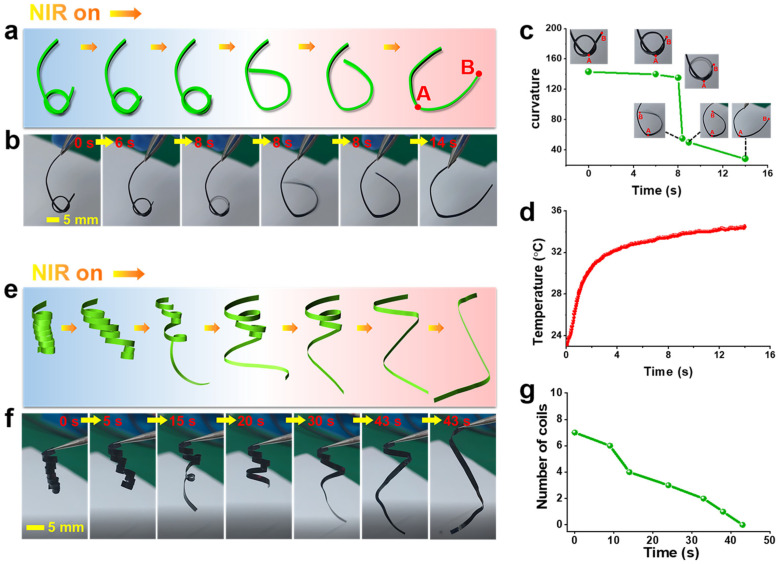
(**a**) Schematic and (**b**) optical photograph documenting the process of MWCNT/LCP s-actuator opening the knot under NIR irradiation (power density: 22.8 mW/mm^2^); change in (**c**) curvature and (**d**) temperature over time for MWCNT/LCP during the process of opening the knot; (**e**) schematic and (**f**) photograph of “spring”-shaped MWCNT/LCP s-actuator rotating and unfolding under NIR irradiation with a power density of 22.8 mW/mm^2^; (**g**) the remaining number of coils over time during the unfolding process of the “spring” under NIR irradiation.

In order to study the intelligent and biomimetic performance of MWCNT/LCP for s-actuator, several s-actuator models and corresponding deformation and motion modes were designed, for instance, tying a knot on the MWCNT/LCP strip with a width of 2.5 mm and then continuously illuminating the knotted ring by NIR with a power density of 22.8 mW/mm^2^ (at room temperature in air). The Video S1 (Supplementary Materials) as well as photographs in Figure 4a,b show the whole process of MWCNT/LCP strip opening the knot driven by NIR. It can be seen from the photographs that the knotted ring began to expand at the 6th second of NIR irradiation and, in the next instant, the ring popped open rapidly, freeing its end from the knot. As NIR continues to irradiate, the entire MWCNT/LCP strip bends into a “U” shape. Video S1 recorded the process of MWCNT/LCP opening the knot, which took only 14 s from the beginning of NIR irradiation to the “U” shape. In order to further study the deformation, two points “A” and “B” were marked on the MWCNT/LCP strip, and their positions are shown in the “U”-shaped diagram in Figure 4a. Quantitative curvature changes of arc AB during the process of opening the knot were calculated and given in Figure 4c, which shows that, under continuous NIR irradiation, the curvature of arc AB gradually decreased from 143.3 to 28.3, with a slow decrease in the first 8 s. In the 8th second, the curvature suddenly dropped from 135 to 50, which is shown as an almost vertical curve in Figure 4c. According to previous reports [30,31,32,33], MWCNTs have brilliant photo-absorption to NIR and efficient thermal conversion performance, resulting in a rapid increase in the temperature of MWCNT/LCP strip under NIR irradiation. Therefore, the liquid crystal to isotropic phase transition occurred, making the movement of LCP molecular chains easy. The temperature recorded synchronously during the MWCNT/LCP knot opening process under NIR irradiation is shown in Figure 4d, from which it can be seen that the temperature of MWCNT/LCP strip sharply increased from room temperature (22 °C) to 31 °C within 2.2 s of irradiation, followed by a slow climbing process, slowly rising to 34.5 °C from 2.2 s to 14 s.

Figure 4e shows the schematic diagram of a “spring”-shaped MWCNT/LCP strip rotating and unfolding under NIR irradiation with a power density of 22.8 mW/mm^2^, and Figure 4f shows the corresponding optical photograph. The “spring”-shaped MWCNT/LCP (denoted as “spring” hereafter) was clamped with tweezers to suspend it vertically (Figure 4f). It can be clearly seen from Video S2 (Supplementary Materials) that, when the NIR spot slowly moves to irradiate each coil of the “spring”, the irradiated coils quickly rotate and unfold. At 43 s of irradiation, all coils have unfolded and the “spring” has been restored to MWCNT/LCP strip. Video S2 records the entire process of “spring” unfolding, during which the remaining number of coils over time is shown in Figure 4g, indicating that, as the irradiation time increases, the remaining number of coils of the “spring” decreases almost linearly.

**Figure 5:**
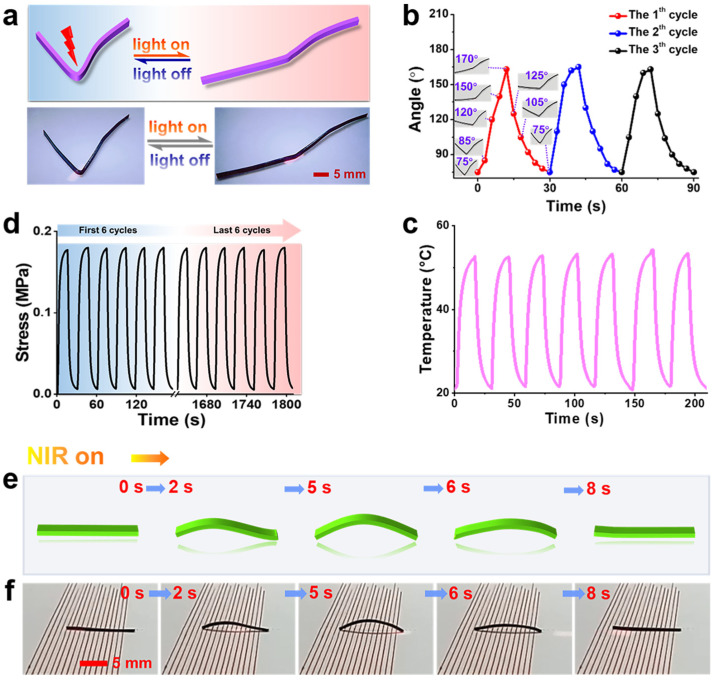
(**a**) Schematic and photographs of the “V”-shaped s-actuator made by MWCNT/LCP that can perform reversible “stretch-restoration” motion under on/off NIR with a power density of 22.8 mW/mm^2^; change in (**b**) angle and (**c**) temperature over time during reversible deformation of “V” s-actuator regulated by on/off NIR; (**d**) actuation stress was measured over 60 cycles for reversible deformation of the “V”-shaped s-actuator, showing almost no attenuation during sustained reversible deformation; (**e**) schematic and (**f**) photographs documenting the process of a 2 wt% MWCNT/LCP strip, which imitates a “caterpillar” walking in a straight line under NIR irradiation with a power density of 22.8 mW/mm^2^.

As shown in Figure 5a, MWCNT/LCP strips with a width of 3.0 mm were premolded into a “V”-shaped s-actuator whose initial angle was 75°. When its corner was continuously illuminated by NIR, the left side of the angle moved counterclockwise until it was approximately on a straight line with the right side. At this point, the angle reaches the maximum, which was 170° and took 13 s. Then, the NIR was off, and the left side of the “V” s-actuator began to slowly reset in an attempt to restore, while a slight recovery occurred on the right. The “V” s-actuator returned to the initial 75 ° within 17 s. The change in angle over time during reversible deformation was measured, as shown in Figure 5b. Figure 5a is a schematic and physical photograph of the “V” s-actuator performing reversible “stretch-restoration” deformation under on/off NIR with a power density of 22.8 mW/mm^2^, and Video S3 (Supplementary Materials) recorded the dynamic cycle of reversible deformation of “V” s-actuator.

Figure 5c shows the real-time temperature response at the corner of the “V” s-actuator during reversible deformation, which demonstrates a rapid, reversible, and cyclic photo-response regulated by cyclic on/off NIR with a power density of 22.8 mW/mm^2^ (at room temperature of 22 °C in air). The peak of temperature rise is 53 ± 1 °C under NIR irradiation. For each NIR cycle, the temperature response is almost the same, and completely matches with the angle changes of the “V” s-actuator (Figure 5b), illustrating that the mechanism of “V” s-actuator deformation is related to its temperature changes dependent on NIR. In fact, the temperature change at the corner of the “V” s-actuator induced by on/off NIR would lead to a reversible phase transition from liquid crystal to isotropic phase of LCP in the nanocomposites at the micro level. In the amorphous isotropic phase (heating up), the molecular chains moved very actively, while they returned to a regular state and were bound in the liquid crystal phase (cooling). Furthermore, the repeatability and stability of reversible deformation of “V” s-actuator were investigated by measuring the actuation stress during its reversible deformation process (Figure 5d). As shown in Figure 5d, the actuation stress exhibits a reversible and cyclic dynamic change and almost without attenuation over 60 cycles of sustained reversible deformation.

MWCNT/LCP could also walk in a straight line like caterpillars in nature. When a 15 × 2 mm^2^ MWCNT/LCP strip (thickness: 0.5 mm) was irradiated with NIR from front to back, its body slowly arched into an arch, pulling the end forward in the process, and, when NIR was off, the arch sank back to a straight line, in which its front section was pushed forward. As shown in Video S4 (Supplementary Materials), when the 2 wt% MWCNT/LCP strip was periodically irradiated from front to back by NIR, it could crawl continuously forward at an average speed of 13 s/mm, and its movement and form were vivid, like living caterpillars. Figure 5e,f are schematic diagrams and photos of the 2 wt% MWCNT/LCP strip completing a continuous forward motion in one cycle of NIR irradiation, respectively. The 4 wt% MWCNT/LCP had also been studied, due to the opposite direction of the static friction (between the middle part and the ground) generated when the tail and head were raised under NIR irradiation (Figure S5), the driving force of forward walking was greatly weakened, which, in turn, gave a very slow movement speed. Details are analyzed in Figure S5. Therefore, 2 wt% MWCNT/LCP has better performance than 4 wt% MWCNT/LCP in mimicking caterpillar linear walking.

In addition, two bionic “insect” models were fabricated using 2 wt% MWCNT/LCP nanocomposites, as shown in the first photo in Figure S6a,b, respectively. Under NIR irradiation, the first “insect” crawled a small distance in 24 s (Figure S6a and Video S5 in Supplementary Materials), and the second one completed a “rolling” motion within 5 s of exposure (Figure S6b and Video S6 in Supplementary Materials). The details of deformation and motion are described in Supplementary Materials.

MWCNT/LCP can not only mimic animal behavior but also plant behavior. Video S7 (Supplementary Materials) records the dynamic process of the MWCNT/LCP “flower bud” model gradually blooming into a “flower”. When the “flower bud” was irradiated by NIR, the three petals began to deform simultaneously (0~2 s), and bloomed quickly until they reached a semi blooming state (the 23th second) when continuously irradiated. After that, the NIR spot could only irradiate a single petal; thus, the spot position could only be moved slowly so that the three petals unfolded one by one. The blooming process and final shape of the MWCNT/LCP s-actuator capable of “flowering” are shown in Figure S7.

In this study, the optimal power density for NIR driving MWCNT/LCP s-actuators deformation and motion is 22.8 mW/mm^2^. In fact, during the initial exploration, four power densities of NIR, namely 18, 22.8, 32, and 40 mW/mm^2^, were adopted. However, the deformation and motion performance of MWCNT/LCP s-actuators in response to the other three kinds of NIR were not satisfactory. Figure S8 shows the typical research results of the other three kinds of NIR-driven s-actuators deformations, mainly manifested as slow deformation and motion rates and small deformation amounts. A large number of experimental results show that 22.8 mW/mm^2^ is the NIR with the best driving effect.

## 4. Conclusions

A series of NIR-driven s-actuators capable of biomimetic deformation and motion have been developed employing a novel MWCNT/LCP nanocomposite film that is easily prepared and fabricated into s-actuators without complicated assembly. By precisely controlling the position and time of NIR irradiation, MWCNT/LCP s-actuators can mimic the motion behavior of animals and plants in nature, accomplishing complex deformation and motion tasks such as opening a knot, “V”-shaped reversible deformation, a “spring” rotating and unfolding, imitating a “caterpillar” walking in a straight line (average speed: 13 s/mm), imitating “insects” crawling and rolling, “flowering”, etc. Deformation and motion are macroscopic manifestations of the active movement of molecular chains, which is attributed to the destruction of rod-shaped mesogenic units caused by the liquid crystal–isotropic phase transition of LCP that stem from the photothermal conversion of MWCNTs. In addition, MWCNT/LCP also has superior flexibility and stretchability (ε = 200%) that are essential for s-actuators. The main research contents and results of this study are summarized in Table 1. Based on these outstanding combined attributes, the MWCNT/LCP and its motion models developed in this study take a significant step forward toward their potential application in soft robotics.

## Figures and Tables

**Table 1:** The main research content and results obtained in this study.

Research Contents	Research Results
Performance of MWCNT/LCP Nanocomposites	thickness: 0.5 ± 0.1 mm stretchability: tensile strain can reach 200% (Figure S4a). outstanding flexibility (Figure 2e and Figure S4b)
Performance of MWCNT/LCP S-actuators	Manufacturing Process: Various soft actuator models can be easily fabricated without complicated assembly. Performance: By precisely controlling the position and time of NIR irradiation, MWCNT/LCP s-actuators can deform and move like creatures in nature, such as opening a knot, “stretch-restoration” reversible deformation of “V”-shape (30 s per cycle), the “spring” rotating and unfolding, imitating a “caterpillar” walking in a straight line (the average speed is 13 s/mm), and an “insect” crawling and rolling, etc.
Mechanism of MWCNT/LCP S-actuators	The macroscopic deformation and motion of MWCNT/LCP s-actuators are attributed to the destruction of rod-shaped mesogenic unit of the LCP at the micro level due to the photothermal effect-regulated liquid crystal–isotropic phase transition in LCP, which makes the movement of the molecular chains more active.

## Data Availability

Data are contained within the article and Supplementary Materials.

## Supplementary Materials

The following Supplementary Materials can be downloaded at: https://www.mdpi.com/article/10.3390/polym17101315/s1, Figure S1: The fourier transform infrared spectroscopy (FTIR) of functionalized MWCNTs, LCP and 2 wt% MWCNT/HEPCP nanocomposite; Figure S2: SEM images of surface and cross-section of MWCNT/LCP films; Figure S3: The photos document the outstanding stretchability (*ɛ* = 200%) and flexibility of the 2 wt% MWCNT/LCP films (thickness: 0.5 mm); Figure S4: Continuous motion of the 4 wt% MWCNT/LCP strip being driven by NIR to “walk” in a straight line; Figure S5: Bionic “insect”-shaped MWCNT/LCP (2 wt%) soft actuators (s-actuator) were driven by NIR to crawl and roll; Figure S6: Photographs of the MWCNT/LCP (2 wt%) “flowering” process under NIR irradiation. Table S1: Characteristic absorption peaks and corresponding functional groups of MWCNT/LCP; Videos S1–S7: MWCNT/LCP s-actuators imitate the behavior of creatures in nature under NIR irradiating, such as opening a knot, “V”-shaped reversible deformation, a “spring” rotating and unfolding, imitating a “caterpillar” walking in a straight line and “insects” crawling and rolling, and “flower” in bloom.
