# Supplementary material for: Biomimetic Soft Actuator with Deformation and Motion Driven by Near-Infrared Light
Source: Polymers (Basel). 2025 May 12;17(10):1315. doi: 10.3390/polym17101315 (PMC12115308; doi:10.3390/polym17101315)
Supplement: Supplementary file 1 [file polymers-17-01315-s001.zip › Supporting Information.pdf]

Supporting Information

Biomimetic Soft Actuator with Deformation and Motion Driven by Near-Infrared Light

Mei Li [1] and Yubai Ma [2,*]

[1] College of Materials Science and Engineering, Chongqing University, Chongqing 400044, China; 15156215827@163.com
[2] School of Chemistry and Chemical Engineering, Chongqing University, Chongqing 400044, China
* Correspondence: mayb168@hotmail.com; Tel.: +86-15156215827

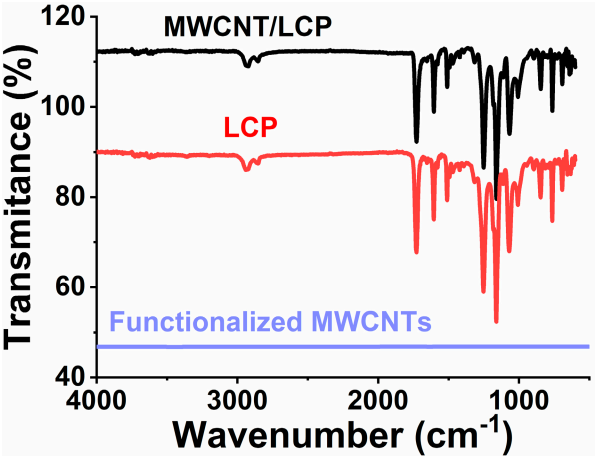

Figure S1. The fourier transform infrared spectroscopy (FTIR) of functionalized MWCNTs, LCP and 2 wt% MWCNT/HEPCP nanocomposite.

FTIR was used to analyze the chemical structure of the liquid crystal polymer (LCP) and multi-walled carbon nanotubes (MWCNT)/LCP nanocomposites. It can be seen from Figure S1 that the characteristic absorption peaks of MWCNT/LCP are almost identical to those of LCP, and their functional groups corresponding to each characteristic absorption peak have been analyzed and listed in Table S1. It should be noted that ν_{as} represents asymmetric stretching vibration of the molecule, ν_s represents symmetric stretching vibration, δ represents bending vibration of the molecule, s represents strong absorption, vs represents very strong absorption, m represents moderate intensity absorption, and w represents weak absorption.

Table S1. Characteristic absorption peaks and corresponding functional groups of MWCNT/LCP.

Wavenumber (cm^{-1})	Functonal Group	Intensity
2929 (ν_{as}), 2854 (ν_s)	CH_2	w
1730 (ν_{as})	C=O	s
1604 (ν), 1507 (ν)	C=C aromatic	m
1470 (δ)	CH_2	w
1316 (δ)	C-N	w

1251 (ν_{as}), 1160 (ν_{as})	C-O-C	vs
1067 (ν_{as}), 1007 (ν_{as})	C-O-C	s
845 (δ)	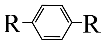	m
763 (δ)	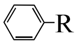	m
692	N-H	w

It could be seen from the Scanning electron microscopy (SEM) images that MWCNTs were uniformly dispersed both on the surface (Figure S2a) and inside (Figure S2b) of the film, forming a dense and uniform network structure that performed photothermal conversion and transfer for the NIR response of MWCNT/LCP.

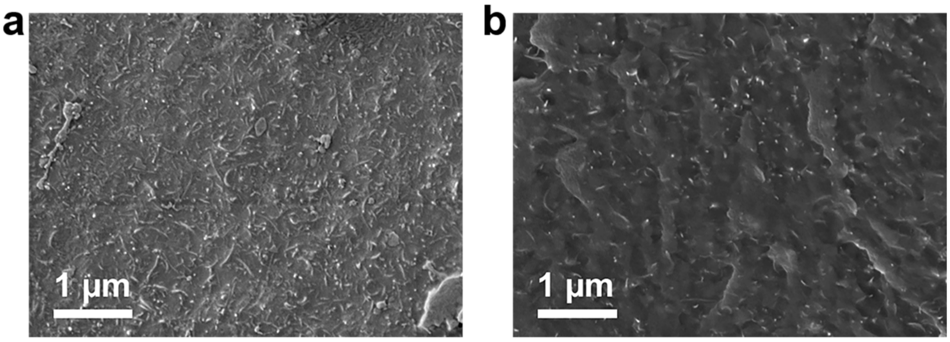

Figure S2. SEM images of (a) surface and (b) cross section of MWCNT/LCP films.

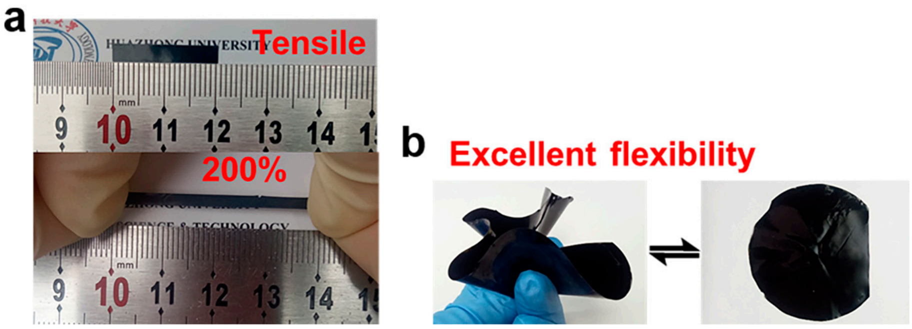

Figure S3. The photos document the outstanding (a) stretchability (ε = 200%) and (b) flexibility of the 2 wt% MWCNT/LCP films (thickness: 0.5 mm).

MWCNT/LCP films can be stretched to a strain of 200% (Figure S3a), can also be molded into any shape under external force, and then can be restored to the original state when the external force disappears (Figure S3b), demonstrating their outstanding stretchability and flexibility.

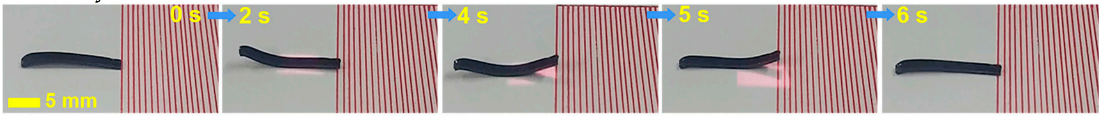

Figure S4. Continuous motion of the 4 wt% MWCNT/LCP strip being driven by NIR to "walk" in a straight line.

4 wt% MWCNT/LCP could also mimic a "worm" walking in a straight line. However, its motion mode was opposite to 2 wt% MWCNT/LCP. As shown in Figure S4, when NIR irradiated the MWCNT/LCP strip from back to front, the middle part of the body is firmly

attached to the ground, while the tail and head lift up successively, forming an inverted arch, and when NIR is off, the tail and head slowly sink down successively, returning to the original state. In this motion mode, the static friction (between the middle part and the ground) generated when the tail and head were lifted was in the opposite direction under NIR irradiated, resulting in a significant weakening of the driving force for forward movement, thereby a very slow movement speed. It can be seen that 2 wt% MWCNT/LCP performs better than 4 wt% MWCNT/LCP in mimicking worm linear walking.

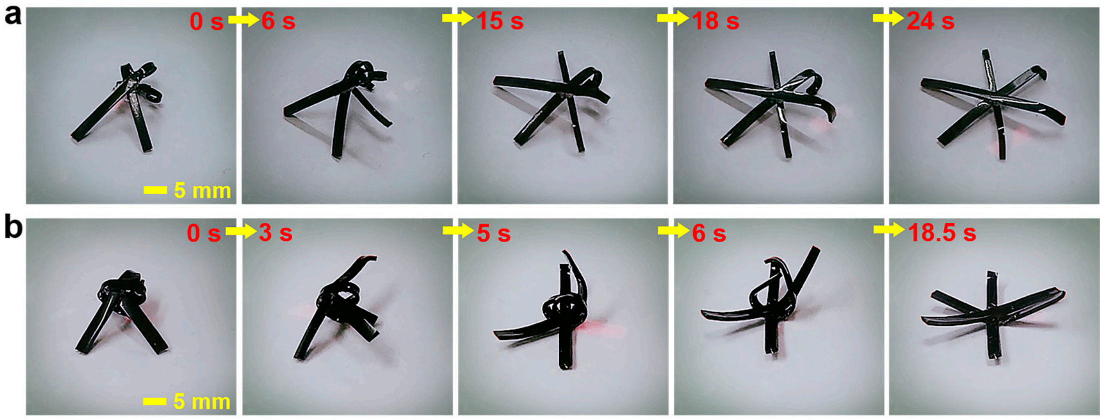

Figure S5. Bionic "insect"-shaped MWCNT/LCP (2 wt%) soft actuator (s-actuator) were driven by NIR to (a) crawling and (b) rolling.

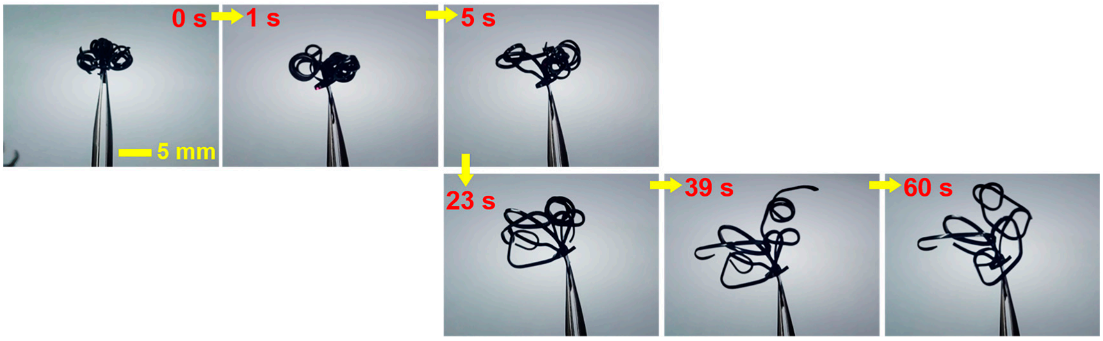

Figure S6. Photographs of the MWCNT/LCP (2 wt%) "flowering" process under NIR irradiation.

Two bionic "insect" models were fabricated using MWCNT/LCP nanocomposites, as shown in the first photo in Figure S5a and b respectively. When the first "insect" (Figure S5a) was irradiated with NIR, it was able to stretch out its four front paws like an insect, showing an effort to crawl forward and moving a small distance within 24 s. The crawling process of the insect was recorded in Figure S5a and Video S5. The second "insect" (Figure S5b) was driven by NIR to perform the "rolling" motion. When its curled front paws were irradiated by NIR, they bounced apart quickly and completed the rolling motion within 5 s, as documented in Figure S5b and Video S6.

Three MWCNT/LCP strips with a width of 1.0 mm were tied together at one end and then all of them were shaped into a tight curl to obtain the "flower bud" shaped model in the first photo in Figure S6. When the "flower bud" was irradiated by NIR, the three petals began to deform simultaneously (0~2 s), and bloomed quickly until they reached a semi blooming state (the 23th second) when continuously irradiated. After that, the NIR spot could only irradiate a single petal, thus the spot position could only be moved slowly so that the three petals unfolded one by one. Video S7 shows the dynamic process of the MWCNT/LCP "flower bud" model gradually blooming into a "flower". The process and final shape of the MWCNT/LCP s-actuator that "blooms" are shown in Figure S6.
